# A Point Mutation in Translation Initiation Factor 2B Leads to a Continuous Hyper Stress State in Oligodendroglial-Derived Cells

**DOI:** 10.1371/journal.pone.0003783

**Published:** 2008-11-21

**Authors:** Liraz Kantor, Dalia Pinchasi, Michelle Mintz, Yetrib Hathout, Adeline Vanderver, Orna Elroy-Stein

**Affiliations:** 1 Department of Cell Research & Immunology, George S. Wise Faculty of Life Sciences, Tel-Aviv University, Tel-Aviv, Israel; 2 Center for Genetic Medicine, Children's National Medical Center, Washington D. C., United States of America; National Institutes of Health, United States of America

## Abstract

**Background:**

Mutations in eukaryotic translation initiation factor 2B (eIF2B) cause Childhood Ataxia with CNS Hypomyelination (CACH), also known as Vanishing White Matter disease (VWM). The disease is manifested by loss of brain myelin upon physiological stress. In a previous study, we showed that fibroblasts isolated from CACH/VWM patients are hypersensitive to pharmacologically-induced endoplasmic reticulum (ER) stress. Since brain cells from affected individuals are not available for research, we wished to assess the effect of eIF2B mutation on oligodendroglial-derived cells.

**Methodology/Principal Findings:**

A rat oligodendroglial-derived cell line was used for a stable knock-down of eIF2B5 followed by stable expression of mutated eIF2B5(R195H) cDNA. In response to a pharmacological ER-stress agent, eIF2B5(R195H) expressing cells exhibited heightened ER-stress response demonstrated by hyper induction of ATF4, GADD34, Bip, PDIA1, PDIA3, PDIA4 and PDIA6 proteins. Moreover, even in the absence of a pharmacological stress agent, eIF2B5(R195H)-expressing cells exhibited high basal levels of ATF4, GADD34 and ER-associated Bip, PDIA1 and PDIA3.

**Significance:**

The data provide evidence that oligodendroglial-derived cells expressing a mutated eIF2B constantly use their stress response mechanism as an adaptation mean in order to survive. The current study is the first to demonstrate the effects of eIF2B5 mutation on ER homeostasis in oligodendroglial-derived cells.

## Introduction

eIF2B is a major evolutionary conserved eukaryotic translation initiation factor. It consists of five different subunits, α, β, γ, δ and ε, (also referred to as subunits 1–5) at a 1∶1 ratio. Subunit 5 is catalytic, whereas subunits 1–4 are regulatory [1]. The eIF2B complex serves as the guanine nucleotide exchange factor of eIF2, another major translation initiation factor that is activated by binding GTP. eIF2-GTP binds and loads the initiator Met-tRNAi onto the small ribosomal subunit, to form the 43S pre-initiation complex that binds to the mRNA. Upon AUG recognition at each round of translation initiation, GTP is hydrolyzed and eIF2-GDP is released. Inactive eIF2-GDP is then recycled by eIF2B to eIF2-GTP, which can serve again to establish another initiation event [2]. eIF2B activity governs the rate of global protein synthesis in the cell. Upon a variety of stress conditions such as starvation, viral infection, oxidative and ER-stress, eIF2 is phosphorylated by one of four kinases (HRI, PKR, GCN2 or PERK) on Ser51 of its alpha subunit, generating a phosphorylated form of eIF2 that functions as a competitive inhibitor of eIF2B. The ratio of eIF-2B to eIF-2 is approximately 0.6 and 0.3 in rat liver and reticulocytes, respectively [3] and 0.3 in rat oligodendroglial-derived cells (Elroy-Stein, unpublished). Therefore, phosphorylation of only part of the total cellular eIF2 could potentially sequester all of the eIF-2B into an inactive eIF2-eIF2B complex. Due to its essential role in protein synthesis under normal and stress conditions, it is surprising that mutations in each eIF2B subunit, rather than being lethal, specifically lead to a neurodegenerative disease in humans. This disease, termed eIF2B-related leukodystrophy, is also known as Childhood Ataxia with CNS Hypomyelination (CACH) or Vanishing White Matter (VWM). The classical form of the disease is characterized by progressive loss of myelin in the CNS, leading to motor and cognitive neurological symptoms that deteriorate upon physiological stress, such as fever and mild head trauma [4], [5]. For obvious reasons, brain glial cells from patients are not available for research. Therefore, cultured primary fibroblasts from patients were used instead, revealing that the eIF2B-mutated fibroblasts are hypersensitive to ER-stress induced by a pharmacological agent [6]. The current study focused on the effect of eIF2B5 mutation on the ER-stress response of an oligodendroglial-derived cell line. The mutation used here (R195H in eIF2B5) is associated with a particularly severe form of the disease, prevalent in the Cree Native American population [7]. The generated oligodendroglial-derived cells expressing eIF2B5(R195H) exhibited heightened ER-stress response demonstrated by hyper-induction of ATF4, GADD34, Bip, PDIA1, PDIA3, PDIA4 and PDIA6 proteins, in response to Thapsigargin, a pharmacological ER-stress agent. The current study provides evidence that oligodendroglial-derived cells are forced to elicit their adaptation capacity in order to survive in the face of a mutation in eIF2B5, since even under normal conditions they express high basal levels of ATF4, GADD34 and ER-associated Bip, PDIA1 and PDIA3.

## Results

**Figure 1:**
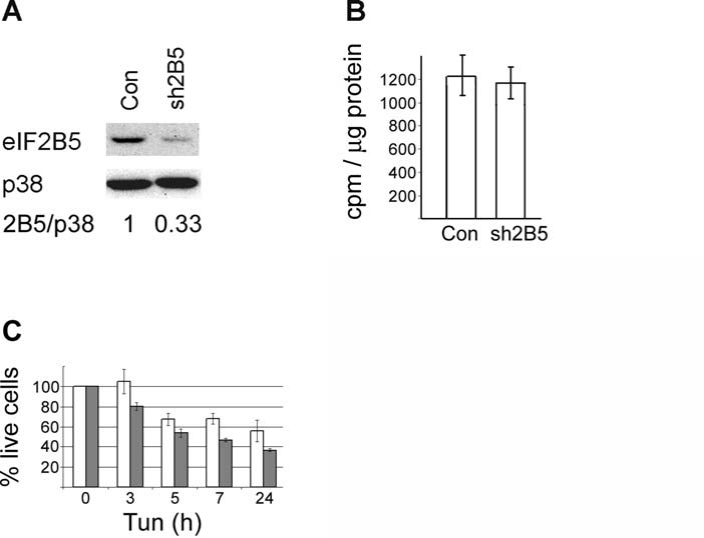
Effect of eIF2B5 down-regulation. A. Identical amounts of total cell protein extracted from DDR1 and sh2B5 cells (stably expressing shRNA against eIF2B5 3′UTR) were subjected to Western blot analysis using antibodies specific for eIF2B5 and p38. B. 5×10^5^ cells were labeled with [^35^S]-Met/Cys mix for 20 minutes followed by protein extraction, TCA-precipitation and scintillation counting of equal amounts of protein. The data represent average of three independent experiments performed in triplicates+/−SE. C. Control (open bars) or sh2B5 cells (dark bars) were incubated with 10 µg/ml Tunicamycin (Tun) for the indicated times, followed by XTT viability assay. Cell viability is expressed as percentages of viable cells grown in Tun-free medium. The data represent average of three independent experiments performed in triplicates+/−SE.

**Figure 2:**
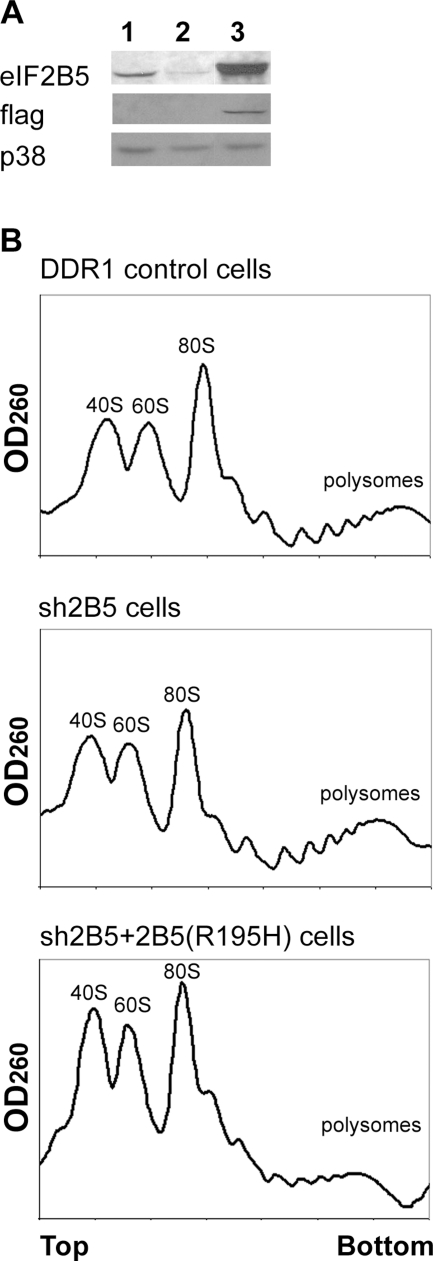
Effect of eIF2B5 overexpression. A. Identical amounts of total cell protein extracted from DDR1, sh2B5 and sh2B5+2B5(R195H) cells (1, 2, or 3, respectively) were subjected to Western blot analysis using antibodies specific for eIF2B5, Flag and p38. B. Polysomal profiles of the indicated cell lines harvested at their logarithmic growth phase. Top and bottom of the sucrose gradients, free ribosomal subunits (40S, 60S), monosomes (80S) and heavy polysomes are indicated.

To study the molecular consequences of eIF2B5 mutation in a cell type that is physiologically relevant to the CNS, we generated a cellular model for CACH/VWM disease using DDR1 cells, a rat cell line of the oligodendroglial lineage. This goal was achieved by a two-step approach. First, the expression of endogenous rat eIF2B5 gene was down-regulated by stable expression of siRNA directed against its 3′UTR. DDR1 stably expressing the pSuperRetro/si2B5-3′UTR plasmid were termed sh2B5 cells. This cell line was then stably transfected with a plasmid expressing a mutated eIF2B5 cDNA lacking the 3′UTR. Fig. 1A shows the lower level of eIF2B5 protein in sh2B5 cells compared to DDR1 controls. Down-regulation of eIF2B5 expression did not exhibit a significant inhibitory effect on global protein synthesis as demonstrated by ^35^S]L-methionine/[^35^S]L-cysteine incorporation rate and polysomal profile analyses (Figs 1B and 2B, sh2B5 cells), indicating that eIF2B5 is expressed in excess amounts by the parental DDR1 cells. To investigate the effect of eIF2B5 down-regulation on cell viability, a time-course experiment was performed in the presence of Tunicamycin (Tun), which blocks the first step of glycoprotein synthesis, thus inhibiting the synthesis of all N-linked glycoproteins and leading to ER-stress. Fig. 1C shows the slight decrease in survival rate of sh2B5 cells compared to DDR1 controls upon 3–24 hr exposure to Tun.

**Figure 3:**
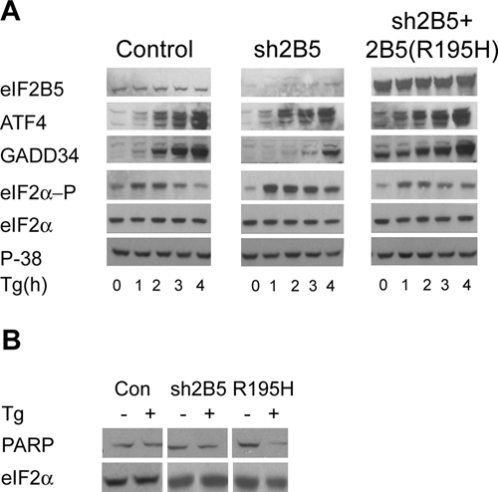
ER-stress response. A. The indicated cells were treated with 3 µM Thapsigargin (Tg) for the indicated time points followed by Western blot analysis using antibodies specific for eIF2B5, ATF4, GADD34, phosphorylated eIF2α (eIF2α -P), total eIF2α and p38. B. Cells were treated with 1 µg/ml Thapsigargin (Tg) for 24 h followed by Western blot analysis using antibodies specific for PARP and total eIF2α.

Since the siRNA was targeted to the 3′UTR of the endogenous eIF2B5, a cDNA of eIF2B5 without the 3′UTR should be resistant to down-regulation by constitutive expression of siRNA in sh2B5 cells. Therefore, sh2B5 cells were stably co-transfected with a vector expressing the Zeocin resistance gene (Zeo) and a vector expressing flag-tagged mutated eIF2B5 cDNA. The R195H mutation of eIF2B5 was chosen because it is associated with a severe form of CACH/VWM disease in human patients [7]. The generated Zeo-resistant stable cell line was termed sh2B5+2B5(R195H). Fig. 2A shows high expression of flag-tagged mutated eIF2B5 protein compared to endogenous eIF2B5 in DDR1 controls and down-regulation thereof in sh2B5 cells. Expression of mutated eIF2B resulted in reduced protein synthesis, as evident from the lower proportions of heavy polysomes in sh2B5+2B5(R195H) cells compared to sh2B5 and DDR1 controls (Fig. 2B). The effect of eIF2B5 mutation on ER-stress response was tested by monitoring ATF4 and GADD34 protein levels at different time points after treatment with Thapsigargin (Tg), which inhibits intracellular calcium pumps and thereby depletes ER calcium stores. In agreement with the kinetics of the unfolded protein response (UPR) [8], [9], an increase in ATF4 and GADD34 protein levels in DDR1 controls and sh2B5 cells was preceded by an increase in eIF2α phosphorylation. However, a more rapid increase in both ATF4 and GADD34 was observed in sh2B5+2B5(R195H) cells, despite similar eIF2α phosphorylation kinetics throughout the three cell lines (Fig. 3A). Also noteworthy is the presence of ATF4 and GADD34 proteins in sh2B5+2B5(R195H) cells even in the absence of a pharmacological ER-stress agent, indicating that mutated eIF2B5 enforces a constant stress state in cells of the oligodendroglial lineage. To further demonstrate the detrimental effect of R195H mutation, the cells were exposed to a low concentration of Tg for 24 h and PARP cleavage was monitored as a marker of apoptosis. Whereas PARP level did not change in DDR1 controls and sh2B5 cells, it was markedly reduced in the mutated cells (Fig. 3B), further demonstrating the increased vulnerability of oligodendroglial-derived cells that express mutated eIF2B5.

**Figure 4:**
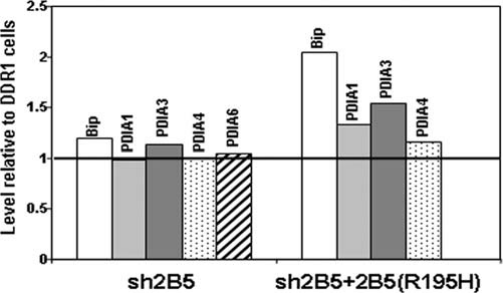
Proteome profiling of ER proteins at baseline. The SILAC methodology followed by mass spectrometry of microsomal preparations was processed as described in Materials and Methods. Untreated labeled DDR1 cells were mixed at a 1∶1 ratio with unlabeled sh2B5 or sh2B5+2B5(R195H) cells. The level of Bip, PDIA1, PDIA3, PDIA4 and PDIA6 relative to DDR1 control cells is shown.

**Figure 5:**
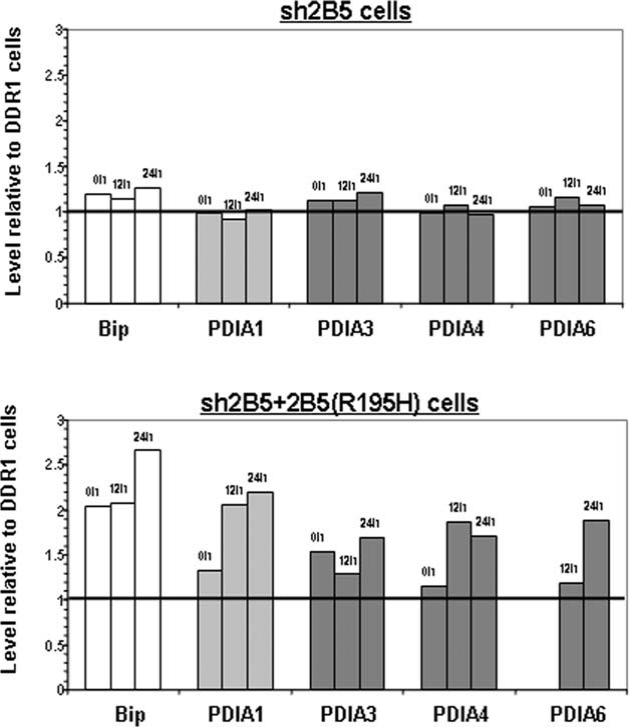
Proteome profiling of ER proteins following stress. The SILAC methodology followed by mass spectrometry of microsomal preparations was processed as described in Materials and Methods. Labeled DDR1 cells and unlabeled sh2B5 or sh2B5+2B5(R195H) were treated with 1 µM Tg for 0, 12 and 24 h. The unlabeled cells at each time point were mixed at a 1∶1 ratio with the labeled DDR1 controls. The level of Bip, PDIA1, PDIA3, PDIA4 and PDIA6 at each time point (except for PDIA6 at baseline in sh2B5+2B5(R195H) cells) relative to DDR1 control cells is shown.

We then determined the ER-associated protein profile under normal and stress conditions using the stable isotope labeling by amino acids in cell culture (SILAC) method. All three cell types were cultured under identical conditions except that the entire protein population of the DDR1 control cells was stably labeled by ^13^C_6_-Arginine and ^15^N_2_, ^13^C_6_-Lysine. All cells were analyzed in the absence of exogenous pharmacological stress as well as following 12 and 24 hours incubation with 1 µM Tg. Pairs were created such that experimental cells and SILAC labeled DDR1 control cells were matched at 0, 12, and 24 hours of Tg incubation, under identical experimental conditions. Cells were harvested and mixed with the corresponding labeled DDR1 control cells at a 1∶1 ratio for subsequent ER fractionation. The ER fractions containing labeled and unlabeled proteins were further separated by SDS-PAGE, followed by trypsin digestion of sliced gel bands. The resulting peptides were analyzed by LC-MS/MS for identification and quantification of proteins. Identified peptides were required to have a matched peptide partner of 6 Da greater for arginine-terminating peptides and 8 Da greater for lysine-containing peptides, in addition to other proper combinations for missed cleavage peptides. Only proteins with two or more identified peptides in at least one data set were retained for analysis. Fig. 4 reflects the proteins status under normal conditions, e.g. shows the basal level of some ER resident proteins in sh2B5 and sh2B5+2B5(R195H) cells compared to DDR1 controls. In sh2B5 cells, the level of Bip (also known as glucose related protein 78, GRP78) and four members of the protein disulfide isomerase family (PDIA1, PDIA3, PDIA4 and PDIA6) was not dramatically changed. However, in sh2B5+2B5(R195H) cells, Bip and PDIA3 were significantly above basal levels as compared to DDR1 control cells (2.0±0.09 and 1.54±0.06 fold up-regulation, respectively). Fig. 5 reflects the ER-stress response, i.e. shows the change in the levels of the above proteins at 12 and 24 hours of mild ER-stress (1 µM Tg, compared to 3 µM Tg used in Fig. 3). In sh2B5 cells, the levels of Bip as well as the four PDI proteins were similar to that of the control cells at each of the three time points, indicating that eIF2B down-regulation did not have a significant effect on the response to long-term mild ER stress. However, although the basal levels of Bip, PDIA1 and PDIA3 were high to begin within sh2B5+2B5(R195H) cells, it further increased compared to DDR1 cells under mild ER stress (2.65±0.06, 2.2±0.03 and 1.7±0.08 fold up-regulation at 24 h; 2.1±0.16 and 2.0±0.01 fold up-regulation at 12 h for Bip and PDIA1, respectively) (Fig. 5). Moreover, the levels of PDIA4 and PDIA6 were also higher than their ER-stress induced levels in control cells (1.7±0.09 and 1.9±0.09 fold up-regulation at 24 h, respectively). The hyper-expression of ER-related molecular chaperons in sh2B5+2B5(R195H) cells under mild stress conditions further demonstrates the remarkable effect of this eIF2B mutation on the sensitivity and vulnerability of cells of the oligodendroglial lineage to ER stress.

## Discussion

The current study demonstrates a connection between a mutation in eIF2B and abnormal ER stress state in glial cells. The ER is an essential cellular compartment for protein synthesis and maturation, and a Ca2+ storage organelle. Interference with Ca2+ homeostasis, inhibition of disulfide bond formation or protein glycosylation, as well as hypoxia and oxidative stress can all result in accumulation of misfolded or unfolded proteins, leading to ER stress. An impaired ER-stress response is associated with neurodegeneration and other diseases [10]. The unfolded protein response (UPR) is a pro-survival adaptive pathway responsible for restoring perturbed ER homeostasis. The ER chaperone Bip is involved in protein folding and assembly and serves as a key guardian for ER disturbances. It monitors the folding status of proteins, thus controlling the activation state of the three UPR transducers PERK, IRE-1 and ATF6. Active PERK phosphorylates eIF2α, thereby leading to inhibition of global translation. ATF4 mRNA is specifically translated under these inhibitory conditions due to regulatory sequences in its 5′-untranslated region. The induced ATF4 protein, together with the products of activated IRE-1 and ATF6, trans-activate the transcription of ER-stress target genes, including a variety of ER chaperones [11]. Bip and other ER-resident quality control proteins, such as the protein disulfide isomerase (PDI) family that catalyzes the formation, cleavage, and rearrangement of disulfide bonds, are induced during ER stress to handle the accumulation of misfolded proteins within the ER [12]–[14]. The comparative proteomic SILAC approach used in this study showed that, in response to induced ER-stress, the ER-resident proteins Bip and PDIs were induced to a higher degree in sh2B5+2B5(R195H) cells compared to control oligodendrocytes (Fig. 5) and primary fibroblasts from two CACH/VWM patients homozygous for the same mutation (Mintz et al., submitted for publication). PERK−/− cells and cells expressing eIF2α(S51A) [15], [16] are more sensitive to agents that perturb protein folding in the ER, implying that reduced level of eIF2-GTP-tRNA^Met^_i_ has an important role in activation of ER chaperones towards adaptation to ER stress. Our data support this notion as they shows that mutation in eIF2B leads to increased levels of ER-chaperones.

In the absence of a pharmacological stress agent, high basal levels of ER-associated Bip, PDIA1 and PDIA3 were detected in eIF2B-mutated oligodendroglial-derived cells (Fig. 4) in contrast to eIF2B-mutated fibroblasts (Mintz et al., submitted for publication). Since cells occasionally experience mild ER stress under normal physiological conditions, the increased basal levels of Bip and PDIs presumably compensate for the occasional increased protein load in the ER. The protective role of Bip in neurodegeneration is supported by several studies [17], [18]. Transient cerebral ischemic pre-activation of the ER-stress response has been shown to be associated with a several fold rise in Bip protein levels and less PERK activation, leading to delayed neuronal cell death [19]. IFN-γ activation of PERK in mature oligodendrocytes was shown to prevent demyelination in a mouse model for multiple sclerosis [20]. Similar to this rationale, increases in the rates of Bip and PDI mRNA synthesis in rat exocrine pancreatic cells precede extensive mRNA expression of secretory proteins induced by glycocorticoid hormones [21]. Neuronal overexpression of PDI has also been shown to promote survival in response to stresses that induce ER dysfunction [22].

Perfect timing of translational recovery along the stress response is crucial for stress resistance [23]. Stress-induced gene expression requires programmed recovery from translational repression, a process induced by GADD34, which recruits protein phosphatase 1 catalytic subunit (PP1c) for removal of the inhibitory phosphate group on eIF2α, thereby reversing the shutoff of protein synthesis [8]. The high basal level of GADD34 in the absence of exogenous stress in sh2B5+2B5(R195H) glial cells (Fig. 3A, time zero) is consistent with the notion that these cells suffer from an intrinsically low threshold of protein synthesis, which is supported by lower proportions of heavy polysomes, as indicated in Fig. 2B. Therefore, the activation of GADD34 as a cellular rescue mechanism may serve to maintain the minimal protein translation rate critical for survival. The induction of GADD34 expression by transient cerebral ischemia in resistant cells, but not in specific vulnerable neurons, is in line with this notion [24].

Although the cell line of the oligodendroglial lineage used in this study do not myelinate nerve axons, the increased levels of proteins responsible for ER quality control under normal conditions strongly suggest that sh2B5+2B5(R195H) cells suffer from elevated burden of occasional ER overload with client proteins. Although their adaptation capacity enables survival even in the face of mutation in eIF2B, their hyper ER-stress response (Figs 3A and 5) suggests that increased ER burden imposes a bona fide threat. The vulnerability of the eIF2B-mutated glial cells to such threat is evident by their propensity to apoptosis (Fig. 3B) albeit a heightened stress response. The consequence of such a feature on actively myelinating oligodendrocytes in the brain may be deleterious. Supporting the above concept is the active unfolded protein response (UPR) state demonstrated in mixed glia of CACH/VWM patients [25], [26].

## Materials and Methods

### Plasmids

Oligoengine™ [27] was employed to design oligonucleotides for long-term gene knockdown following cloning into the pSuperRetro vector (Invitrogen). The designed oligonucleotides 5′GATCCCCCGGAAGTTGCAACTACAGTTTCAAGAGAACTGTAGTTGCAACTTCCGTTTTTGGAAA3′ and 5′AGCTTTTCCAAAAACGGAAGTTGCAACTACAGTTCTCTTGAAACTGTAGTTGCAACTTCCGGGG3′ were annealed to generate a fragment that matches 19 nucleotides region within the 3′UTR of rat eIF2B5 cDNA (region 2361–2379, NM_138866). The fragment was cloned into HindIII and BglII sites of pSuperRetro under the RNA pol1 promoter, to generate pSuperRetro/si2B5-3′UTR. The oligonucleotides 5′CAGCTAGATCTTCACTTGTCCCAGCCC3′ and 5′CCGTGAGAGAAGCTTATGGCGGCC3′ were used to amplify the 2.2 Kbp human eIF2B5 cDNA followed by its cloning into BglII and HindIII sites of pCMV Flag2 (Sigma) to generate pNflag-2B5(wt) which expresses N-terminal Flag-tagged human eIF2B5 from the CMV promoter. This plasmid was used as template for further PCR reactions: the first product was generated using oligonucleotide primers *2b5 Mut sense2* (5′CGGTAGGCGTGTACGGTGGGAG3′) and *MutG584A antisense* (5′CGTGGCAATGAGTTGGGTGGC3′); the second product was generated using oligonucleotide primers *2b5 Mut antisense* (5′TCCTCTGCATCTGGAGGGTGCA3′) and *MutG584A sense* (5′GCCACCCAACTCATTGCCACG3′). The two PCR products were then annealed and used as template for a third PCR using *2b5 Mut sense2* and *2b5 Mut antisense* primers to generate a fragment harboring the human eIF2B5 cDNA with the G584A point mutation. This fragment was then cloned into the ApaI - HindIII 5.57 Kbp fragment of pNflag-2B5(wt) to generate pNflag-2B5(R195H).

### Cells and stable transfections

DDR1 rat oligodendrocytes cell line [28] kindly obtained from Bernard Attali (Tel Aviv University) was grown on Dulbeco Modified Eagles Medium (DMEM, Biological Industries) supplemented with 10% Fetal Calf Serum, 100 U/ml penicillin and 100 µg/ml streptomycin (Biological Industries). DDR1 cells were stably transfected using the standard calcium-phosphate precipitation technique. To generate control and sh2B5 cells, DDR1 cells were transfected with the plasmids pSuperRetro (Invitrogen) or pSuperRetro/si2B5-3′UTR, respectively, and selected using medium containing 1 µg/ml puromycin. To generate sh2B5+2B5(R195H) cells stably overexpressing the mutated human eIF2B5 on the background of endogenous rat eIF2B5 knocked-down, sh2B5 cells were co-transfected with pCDNA4/To (Invitrogen) and pNflag-2B5(R195H) followed by selection for Zeocine resistance in medium containing 100 µg/ml Zeocine and 1 µg/ml puromycin.

### Protein synthesis rate and polysomal profiles analysis

6×10^5^ cells were plated in 60 mm plates and labeled for 20 min with 20 µCi/ ml ^35^S-L-methionine, ^35^S-L-cysteine mix (NEN, #NEG072) in their growth medium, followed by two washes with cold PBS. Extraction and processing of labeled proteins were performed as previously described [29]. Polysomal profiles analyses were performed according to Sivan et al [30] using extract prepared from 2×10^7^ cells (30 OD260 nm units) per each sucrose gradient. Teledyne ISCO UA-6 UA6 Absorbance Detector was used for analysis.

### XTT viability Assays

5×10^3^ cells were plated in each well of a 96-well plate. Cells were seeded in triplicates. The following day, the medium was replaced with fresh medium containing 10 µg/ml Tunicamycin (Tun). The plates were incubated for different time periods before addition of 50 µl of 2,3-bis[2-methoxy-4-nitro-5-sulfophenyl]-2*H*-tetrazolium-5-carboxanilide inner salt (XTT) reaction solution (Biological Industries) to each well; Following incubation of 2 hr the optical density was measured at 450 nm. Viability was calculated relative to cells incubated in the absence of Tun.

### Antibodies

The following antibodies were used: anti p38 (Sigma, M0800), anti FLAG M2 (Sigma, F3165), anti mouse ATF4 and GADD34 (gifts from David Ron, NYU), anti eIF2α phosphorylated at serine 51 (Research Genetics, Inc.), anti human eIF2B5 (Santa Cruz #28854) and monoclonal antibody specific for eIF2α [31].

### SILAC Technique and ER stress induction

DDR1 control cells were seeded in 75 cm^2^ flasks and subcultured in custom-made DMEM medium (Atlanta Biologicals, Lawrenceville, GA) where Arg and Lys were replaced by ^13^C_6_-Arg (147.5 µg/ mL), and ^15^N_2_, ^13^C_6_-Lys (91.25 µg/mL) (Cambridge Isotope Laboratories, Inc., Andover, MA). Cellular proteins were fully labeled after incubation for at least 7 cell doublings. In parallel, the same amounts of experimental cells (sh2B5 and sh2B5+2B5(R195H)) were grown in unlabeled DMEM medium. Both the unlabeled experimental cells and the labeled DDR1 control cells were treated with thapsigargin (final concentration 1 µM) (Tg). At time points 0, 12 and 24 hours cells were harvested followed by mixing of each experimental unlabeled cells at 1∶1 ratio with the labeled DDR1 controls for subsequent subcellular fractionation.

### Preparation of microsomal fractions, mass spectrometry analysis and database search

This experiment was performed as thoroughly described by Mintz et al. [14]. Briefly, ER fractions containing labeled and unlabeled proteins were further separated by SDS-PAGE. Thereafter the resulting gel bands were sliced, digested with trypsin and analyzed by LC-MS/MS using LTQ instrument (Thermo Fisher Scientific, Waltham, MA) coupled to Dionex LC-Packing system (Sunnyvale, CA). Protein identification was performed and filtered using BioWorks 3.1 software (Thermo Fisher Scientific). Each file was searched against a subset of the SwissProt database. Full Sequest search results, prior to any filtration, were loaded into ZoomQuant Software. Identified peptides were required to have a matched peptide partner 6 Da greater for arginine terminating peptides, 8 Da greater for lysine containing peptides and other proper combinations for missed cleavage peptides. ZoomQuant software was used to determine protein ratios between ^13^C_6_, Arg/, ^15^N_2_, ^13^C_6_-Lys labeled and unlabeled cells. Peptide ratios were normalized to the median expression in each time point of the experimental group. An average ratio with standard deviation was determined using the multiple peptides detected per protein. Only proteins with two or more identified peptides in at least one data set were retained for analysis. The data was analyzed using GeneSpring GX Analysis Platform (Agilent technologies Inc, SantaClara, Ca). A two sided p-value or z-score was generated for each data point. Proteins with a z-score<0.005 were retained for further analysis. Fold changes of specific proteins were compared at the different time points between the various cells.
